# Venous thromboembolism in COVID-19: A systematic review and meta-analysis

**DOI:** 10.1177/1358863X21995566

**Published:** 2021-04-04

**Authors:** Anastasios Kollias, Konstantinos G Kyriakoulis, Styliani Lagou, Evangelos Kontopantelis, George S Stergiou, Konstantinos Syrigos

**Affiliations:** 1 Third Department of Medicine, National and Kapodistrian University of Athens, School of Medicine, Sotiria Hospital, Athens, Greece; 2 Division of Informatics, Imaging and Data Sciences, Faculty of Biology, Medicine and Health, University of Manchester, Manchester, UK; 3 National Institute for Health Research, School for Primary Care Research, University of Manchester, Manchester, UK

**Keywords:** deep vein thrombosis (DVT), prevalence, pulmonary embolism (PE), SARS-CoV-2

## Abstract

Severe coronavirus disease 2019 (COVID-19) is associated with increased risk of venous thromboembolism events (VTE). This study performed a systematic review in PubMed/EMBASE of studies reporting the prevalence of VTE in patients with COVID-19 who were totally screened/assessed for deep vein thrombosis (DVT) and/or for pulmonary embolism (PE). Among 47 candidate studies (*n* = 6459; 33 in Europe), 17 studies (*n* = 3973; weighted age 63.0 years, males 60%, intensive care unit (ICU) 16%) reported the prevalence of PE with a pooled estimate of 32% (95% CI: 25, 40%), and 32 studies (*n* = 2552; weighted age 62.6 years, males 57%, ICU 49%) reported the prevalence of DVT with a pooled estimate of 27% (95% CI: 21, 34%). A total of 36 studies reported the use of at least prophylactic antithrombotic treatment in the majority of their patients. Meta-regression analysis showed that the prevalence of VTE was higher across studies with a higher percentage of ICU patients and higher study population mean D-dimer values, and lower in studies with mixed dosing of anticoagulation in ⩾ 50% of the population compared to studies with standard prophylactic dosing of anticoagulation in < 50% of the population. The pooled odds ratio for death in patients with COVID-19 and VTE versus those without VTE (17 studies, *n* = 2882) was 2.1 (95% CI: 1.2, 3.6). Hospitalized patients with severe COVID-19 are at high VTE risk despite prophylactic anticoagulation. Further research should investigate the individualized VTE risk of patients with COVID-19 and the optimal preventive antithrombotic therapy. **PROSPERO Registration No.: CRD42020185543.**

## Introduction

Although coronavirus disease 2019 (COVID-19) has been identified mainly as a viral respiratory tract infection, it has become evident that several complications render a systematic approach to this new infectious disease necessary. Emerging evidence shows that severe COVID-19 is often complicated with coagulopathy, which has prothrombotic effects resulting in high risk of venous thromboembolism events (VTE) and mortality.^1–3^ However, it appears that there is a significant heterogeneity in the observed VTE phenotypes (isolated deep vein thrombosis (DVT), isolated pulmonary embolism (PE)/thrombosis, combined DVT and PE)^2^ and the prevalence of VTE among screened patients remains understudied.

Moreover, preliminary evidence suggests that anticoagulant therapy might provide a survival benefit in patients with severe COVID-19.^4,5^ This issue is being increasingly recognized by international societies that strongly recommend the use of thromboprophylaxis in all hospitalized patients.^6–10^

This study aimed to review the current evidence regarding the prevalence of VTE in patients with COVID-19 screened/assessed with lower limb ultrasonography or computed tomography pulmonary angiography.

## Materials and methods

### Data sources and searches

This study protocol was registered in PROSPERO; No.: CRD42020185543.

A systematic literature search of PubMed and EMBASE databases was performed in line with the PRISMA recommendations (www.prisma-statement.org) independently by three investigators (AK, KGK, SL) using the following search keywords: (‘coronavirus 2019’ OR ‘2019-nCoV’ OR ‘SARS-CoV-2’ OR ‘COVID-19’) AND (thrombotic OR thrombosis OR ‘deep vein’ OR ‘pulmonary embolism’ OR thromboemboli*) until September 30, 2020. Articles were also selected from references of relevant articles, by searching in journals’ websites and by hand search. Disagreements were resolved by consensus with a senior author (AK).

### Study selection

Eligible studies were full-text articles in English that: (i) reported the prevalence of PE and/or DVT in patients with COVID-19; and (ii) performed screening/assessment in the total sample for DVT (lower limb ultrasonography) or were focused on patients with suspicion for PE (whole study population subjected to computed tomography pulmonary angiography). Case reports and case series studies with ⩽ 10 patients were excluded. The primary endpoint of this analysis was the pooled estimate of PE and DVT prevalence. The secondary endpoint included the pooled estimate of odds ratio for death in patients with COVID-19 with VTE versus non-VTE.

### Data extraction and risk of bias assessment

Three investigators extracted independently data concerning study design, main characteristics of included populations and data regarding primary and secondary endpoints from included studies where available. The risk of bias was assessed using the Joanna Briggs Institute’s ‘Critical Appraisal Checklist for Analytical Cross Sectional Studies’.^11^

### Data synthesis and analysis

A pooled prevalence estimate was calculated for each outcome, using the numerators and denominators reported and a Freeman–Tukey arcsine transformation^12^ with the metaan command in Stata.^13^ Heterogeneity in the meta-analyzed estimates was quantified using the I^2^ statistic.^14^ A random effects model was used, and we opted for a nonparametric bootstrapped DerSimonian–Laird approach.^15,16^ The pooled estimates were back-transformed to percentages and are reported as such in forest plots. Poisson regression models were used to examine associations and potential determinants of high heterogeneity in the primary outcome, in a meta-regression setting. The covariates of interest in these analyses were: age, percentage of male patients, percentage of patients in an intensive care unit (ICU), antithrombotic treatment characteristics (none, prophylaxis in < 50% of subjects, prophylaxis in ⩾ 50% of subjects, prophylaxis and higher doses in ⩾ 50% of subjects), mean D-dimer values of the examined sample, and quality of included studies. Meta-regression bubble plots were obtained to further examine the association between VTE prevalence and displaying the Poisson model regression line over study observations. Since this was a meta-analysis of prevalence values, publication bias could not be assessed through tests or funnel plots. Odds ratios for death in VTE versus non-VTE patients were calculated using appropriate formulas.^17^ Odds ratios and 95% CI values were logarithmically transformed and SEs were calculated from these values and used in the analysis. Mean values of subgroups were combined where feasible (i.e. when separate values were given for males/females).^18^ Median values were converted to mean values.^19^ Missing information about study population characteristics (i.e. age, percentage of males, percentage of patients in ICU, thromboprophylaxis details, D-dimer values, overlapping populations with other studies, etc.) was retrieved through personal communication with the corresponding authors where possible. An alpha level of 5% was used.

Analyses were performed using Stata Statistical Software, Release 16 (StataCorp LLC, College Station, TX, USA).

## Results

**Figure 1.**
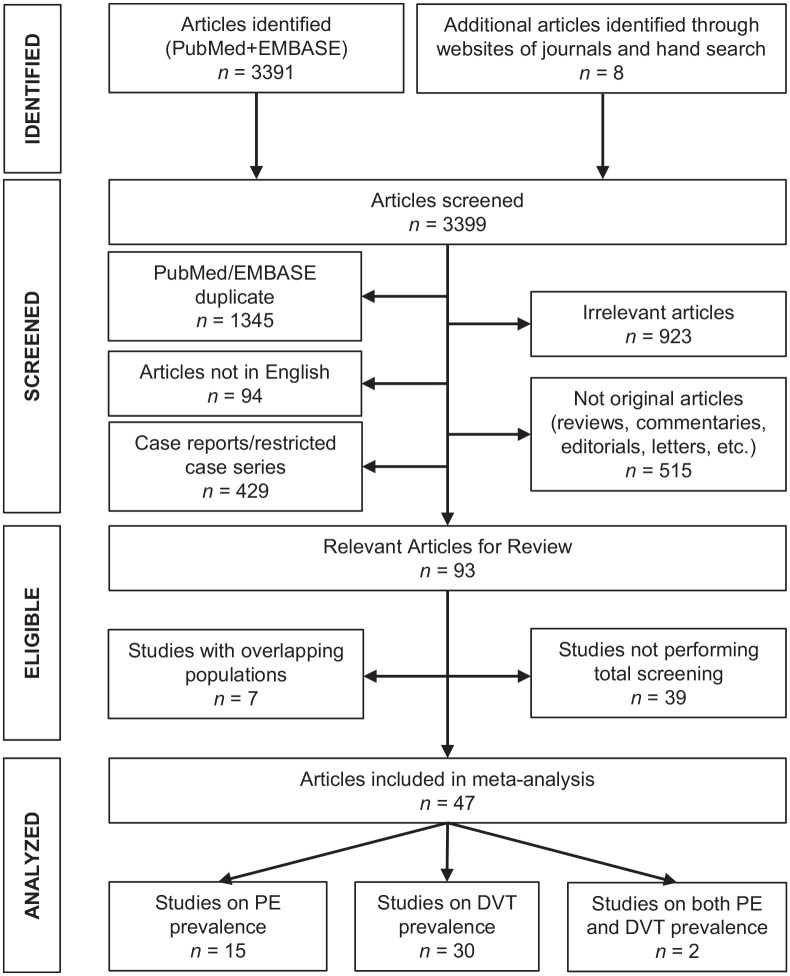
Preferred reporting items for systematic reviews and meta-analyses (PRISMA) flow chart for the selection of studies.

Among 3399 initially identified articles, 47 studies fulfilled the inclusion criteria and were included in the systematic review (Figure 1). The main characteristics of these studies are shown in Table 1.^20–66^

**Table 1.** Main characteristics and findings of studies.

Study	Setting, country	*n*	ICU (%)	Male (%)	Age, years mean ± SD	PCR-based COVID-19 diagnosis (%)	Event for which all patients were evaluated	Symptomatic (%)	Anticoagulation treatment (%)	Prevalence (%)	
										PE	DVT
Ventura-Díaz et al.^20^	Radiology Dept, Spain	242	NR	62	66 ± 15	NR	PE	100	NR	30	
Avruscio et al.^21^	General ward, ICU, Italy	85	48	72	67 ± 13	100	DVT	28	Prophylactic 69 Intermediate 31		42
Longhitano et al.^22^	General ward, ICU, Italy	74	24	59	69 ± 15	NR	DVT	NR	Prophylactic 37 Intermediate 31 Therapeutic 32		16
Pavoni et al.^23^	ICU, Italy	42	100	64	64 ± 12	100	DVT	NR	Intermediate 52 Therapeutic 48		31
Dujardin et al.^24^	ICU, The Netherlands	127	100	77	62 ± 11	100	DVT	NR	Prophylactic 100		21
Espallargas et al.^25^	Radiology Dept, Spain	47	49	64	64 ± 14	100	PE	100	Prophylactic 38 Intermediate 36 Therapeutic 2	34	
Mueller-Peltzer et al.^26^	ICU, Germany	16	100	69	62 ± 8	NR	PE	100	Therapeutic 25	56	
Ramadan et al.^27^	Emergency Dept, general ward, ICU, USA	367	NR	62	61 ± NR	NR	PE	NR	NR	26	
Torres-Machorro et al.^28^	ICU, Mexico	30	100	77	57 ± 33	NR	DVT	0	Intermediate 43 Therapeutic 57		30
Jimenez-Guiu et al.^29^	General ward, Spain	57	0	51	71 ± 13	100	DVT	2	Prophylactic 65 Intermediate 21 Therapeutic 14		11
Mouhat et al.^30^	General ward, ICU, France	162	42	67	66 ± 13	100	PE	100	Prophylactic or Therapeutic 87	27	
Yu et al.^31^	Radiology Dept, China	142	58	57	62 ± 12	NR	DVT	NR	None 74		35
Alonso-Fernández et al.^32^	General ward, ICU, Spain	30	37	63	64 ± 12	100	PE	NR	Prophylactic 87	50	
Giorgi-Pierfranceschi et al.^33^	General ward, Italy	66	0	70	72 ± 11	100	DVT	0	Prophylactic 80 Intermediate 14		14
Le Jeune et al.^34^	General ward, France	42	0	55	65 ± 19	79	DVT	0	Prophylactic 59 Intermediate 24 Therapeutic 17		19
Ierardi et al.^35^	ICU, Italy	234	100	30	62 ± 14	NR	DVT	0	Prophylactic or higher doses 100		11
Alharthy et al.^36^	ICU, Saudi Arabia	89	100	84	43 ± 16	100	DVT	NR	Prophylactic 100		17
Pizzolo et al.^37^	General ward, Italy	43	0	67	65 ± 22	NR	DVT	0	Prophylactic 100		28
Cho et al.^38^	General ward, ICU, USA	158	58	54	67 ± 15	100	DVT	NR	Prophylactic > 90		33
Monfardini et al.^39^	General ward, ICU, Italy	34	32	76	62 ± 9	100	PE DVT	100	Patients with PE Prophylactic 31	76	12
Freund et al.^40^	Emergency Dept, France	974	0	59	61 ± 19	62	PE	100	NR	15	
Chen et al.^41^	Radiology Dept, China	25	NR	60	64 ± 11	60	PE	100	Therapeutic 80	40	
Longchamp et al.^42^	ICU, Switzerland	25	100	64	68 ± 11	100	DVT	24	Prophylactic 100		24
Whyte et al.^43^	General ward, ICU, UK	214	36	60	61 ± 2	NR	PE	100	Prophylactic 100	37	
Marone et al.^44^	Vascular Units, Italy	101	27	58	70 ± 10	NR	DVT	100	Patients with DVT Prophylactic > 90		42
Fauvel et al.^45^	General ward, ICU, France	1240	15	58	64 ± 17	91	PE	100	Prophylactic 63 Intermediate 8 Therapeutic 11	8	
Santoliquido et al.^46^	General ward, Italy	84	0	73	68 ± 14	100	DVT	2	Prophylactic 100		12
Trigonis et al.^47^	ICU, USA	45	100	NR	61 ± 15	NR	DVT	NR	Prophylactic 38 Intermediate 53		42
Larsen et al.^48^	General ward, ICU, Reunion Island	35	11	77	67 ± 17	100	PE	NR	Prophylactic 80	14	
Chen et al.^49^	ICU, China	88	100	61	63 ± 12	NR	DVT	13	Prophylactic 100		45
Koleilat et al.^50^	General ward, USA	135	0	53	63 ± 15	100	DVT	NR	None 19 Prophylactic 63 Therapeutic 18		13
Bavaro et al.^51^	General ward, ICU, Italy	20	30	40	65 ± 23	NR	PE	NR	Prophylactic 85	40	
Grandmaison et al.^52^	General ward, ICU, Switzerland	58	50	NR	ICU patients 62 ± 31	100	DVT	NR	Prophylactic 100		36
Mazzaccaro et al.^53^	General ward, Italy	32	0	72	69 ± 12	100	PE DVT	NR	Prophylactic or therapeutic 100	66	3
Gervaise et al.^54^	Radiology Dept, France	72	NR	75	62 ± 18	80	PE	100	NR	18	
Voicu et al.^55^	ICU, France	56	100	74	60 ± 12	NR	DVT	NR	Prophylactic 100		46
Nahum et al.^56^	ICU, France	34	100	74	62 ± 9	76	DVT	NR	Prophylactic 100		79
Artifoni et al.^57^	General ward, France	71	0	61	62 ± 22	NR	DVT	3	Prophylactic 100		21
Zhang et al.^58^	General ward, China	143	0	52	63 ± 14	NR	DVT	NR	Prophylactic 37		46
Ren et al.^59^	ICU, China	48	100	54	71 ± 14	NR	DVT	NR	Prophylactic 98		85
Poyiadji et al.^60^	Radiology Dept, USA	328	25	46	61 ± 16	100	PE	NR	Prophylactic 37	22	
Demelo-Rodríguez et al.^61^	General ward, Spain	156	0	65	68 ± 15	85	DVT	0	Prophylactic 98		15
Bompard et al.^62^	Radiology Dept, France	135	18	70	65 ± 17	NR	PE	100	ICU patients Prophylactic or intermediate 100	24	
Criel et al.^63^	General ward, ICU, Belgium	82	37	59	64 ± 13	NR	DVT	0	Prophylactic or intermediate 95		7
Cattaneo et al.^64^	General ward, Italy	64	0	55	68 ± 14	NR	DVT	0	Prophylactic 100		0
Llitjos et al.^65^	ICU, France	26	100	77	65 ± 18	100	DVT	NR	Prophylactic 31 Therapeutic 69		54
Cui et al.^66^	ICU, China	81	100	46	60 ± 14	100	DVT	NR	None		25

COVID-19, coronavirus disease 2019; DVT, deep vein thrombosis; ICU, intensive care unit; NR, not reported; PCR, polymerase chain reaction; PE, pulmonary embolism.

**Figure 2.**
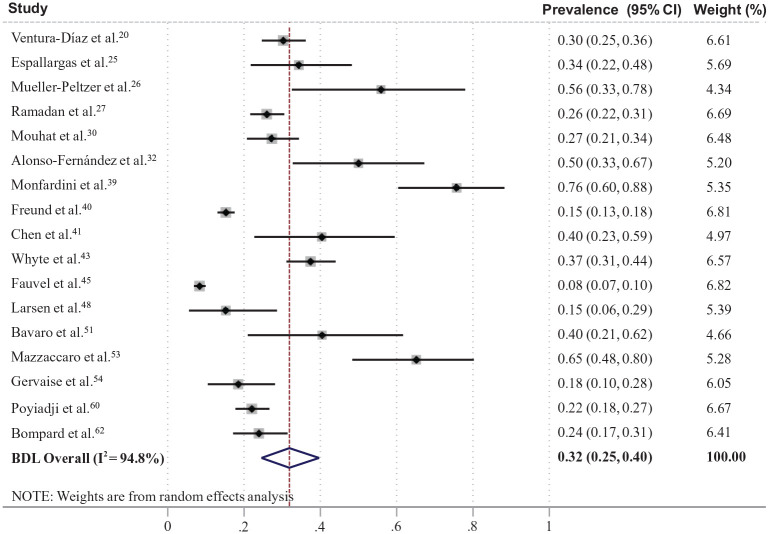
Forest plot of prevalence of pulmonary embolism in patients with coronavirus disease (COVID-19). BDL, Bootstrapped DerSimonian-Laird’ model.

**Figure 3.**
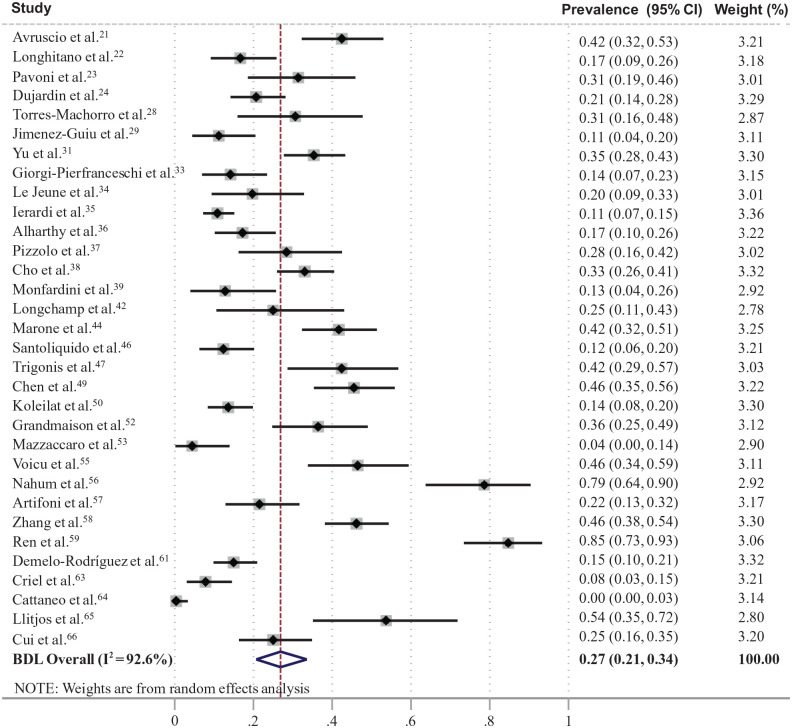
Forest plot of prevalence of deep vein thrombosis in patients with coronavirus disease (COVID-19). BDL, Bootstrapped DerSimonian-Laird’ model.

A total of 47 studies (*n* = 6459; 33 in Europe) reported the prevalence of VTE in totally screened/assessed patients with COVID-19.^20–66^ Among them, 17 studies (*n* = 3973; weighted age 63.0 years, males 60%, ICU 16%) reported the prevalence of PE with a pooled estimate of 32% (95% CI: 25, 40%) (Figure 2),^20,25–27,30,32,39–41,43,45,48,51,53,54,60,62^ and 32 studies (*n* = 2552; weighted age 62.6 years, males 57%, ICU 49%) reported the prevalence of DVT with a pooled estimate of 27% (95% CI: 21, 34%) (Figure 3).^21–24,28,29,31,33–39,42,44,46,47,49,50,52,53,55–59,61,63–66^ A total of 36 studies reported the use of at least prophylactic antithrombotic treatment in the majority of their patients (Table 1).^21–25,28–30,32–38,41–43,45–53,55–57,59,61–65^ The assessment of the risk of bias is presented in online Supplementary Figure S1. In plots of prevalence versus study sample size, there was a trend for higher PE prevalence in smaller studies, but there was no apparent trend in DVT prevalence (online Supplementary Figure S2).

Meta-regression analysis did not reveal any significant associations between mean age, percentage of males, or quality of the included studies and the prevalence of PE/DVT. However, the prevalence of PE was higher across studies with higher mean D-dimer values (prevalence ratio 1.3 per 1000 ng/mL increase; 95% CI: 1.11, 1.50, *p* = 0.002) and higher percentage of ICU patients (1.02 per 1% increase; 95% CI: 1.01, 1.03, *p* < 0.001). In addition, prevalence of DVT was higher across studies with higher mean D-dimer values (1.04 per 1000 ng/mL increase; 95% CI: 1.01, 1.07, *p* = 0.022) and lower in studies with mixed dosing of anticoagulation in ⩾ 50% of the population compared to studies with standard prophylactic dosing of anticoagulation in < 50% of the population (0.49; 95% CI: 0.31, 0.78, *p* = 0.003). Meta-regression bubble plots for noncategorical variables are shown in online Supplementary Figure S3. The above-mentioned estimates regarding the associations of PE prevalence were almost identical when a small study outlier was removed (online Supplementary Figure S4).

**Figure 4.**
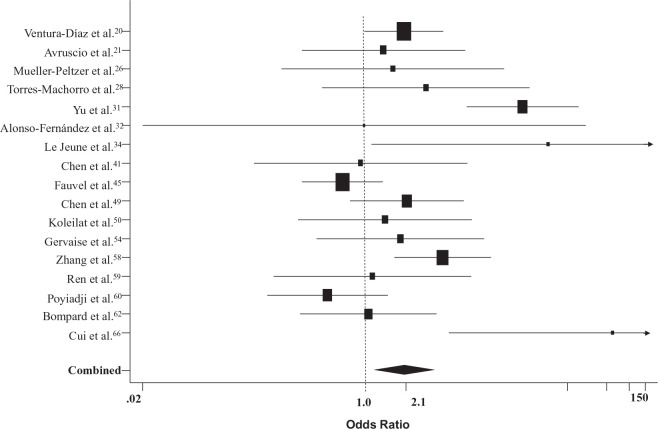
Forest plot of odds ratios for death in patients with COVID-19 and VTE versus those without VTE. COVID-19, coronavirus disease 2019; VTE, venous thromboembolism.

The pooled odds ratio for death in patients with COVID-19 and VTE versus those without VTE (17 studies, *n* = 2882)^20,21,26,28,31,32,34,41,45,49,50,54,58–60,62,66^ was 2.1 (95% CI: 1.2, 3.6) (Figure 4).

## Discussion

The main findings of this analysis were the following: (i) the overall prevalence of PE/DVT in hospitalized patients with COVID-19 subjected to assessment was about 30% but with considerable observed heterogeneity; (ii) VTE prevalence was high, even in patients receiving thromboprophylaxis, and appeared to be higher in studies with < 50% of patients anticoagulated; and (iii) patients with COVID-19 and VTE compared to those without VTE had higher risk for death.

It is well recognized that all hospitalized patients with acute medical illness are at high VTE risk. Critically ill patients admitted to ICUs are at very high VTE risk because of ICU-specific risk factors (immobilization, sedation, vasopressors or central venous catheters), but also individual patient-related risk factors (age, obesity, immobilization, history of personal or familial VTE, cancer, sepsis, respiratory or heart failure, pregnancy, stroke, trauma, or recent surgery).^1,2,67^ Thus, all hospitalized patients, and especially those in ICUs, are routinely assessed for VTE risk and often administered thromboprophylaxis.

At present, whether COVID-19 is associated with a higher VTE risk than other infections remains unclear. Initial case reports of VTE events in patients with COVID-19 were followed by case series studies, mainly conducted in an ICU setting, and showed high VTE prevalence, particularly in patients with severe COVID-19.^20–66,68^ It has been suggested that SARS-Cov-2 in severe forms of the disease induces an excessive inflammatory state via cytokine storm combined with endothelial injury and pulmonary vascular microthrombosis, which could considerably increase the risk for VTE, mainly PE.^1–10^ In recent autopsy studies, it has been found that the lungs of the infected patients are characterized by a widespread thrombosis with microangiopathy, whereas a high incidence of DVT has been recognized with PE being identified as a direct cause of death.^69,70^

This review of the current evidence indicates that the course of hospitalized patients with COVID-19 is complicated with DVT/PE in about 30% of cases, irrespective that most of them have received thromboprophylaxis. Unfortunately, studies providing direct head-to-head comparison and using the same assessment methodology between patients with COVID-19 and patients hospitalized for other reasons in terms of VTE prevalence are lacking. However, data from two studies that compared patients with COVID-19 and other (non-COVID-19) patients hospitalized in the same ICU but at different time points, showed higher prevalence of VTE in COVID-19.^71,72^ These data might support the notion that COVID-19 is associated with a higher risk of thrombosis than other diseases requiring ICU admission but future, well-designed studies should confirm this finding.

Another important finding was that patients with COVID-19 and VTE had a higher risk for death compared to those without VTE. Unfortunately, the exact cause of death (all-cause versus VTE-related) in these patients has not been reported in the included studies. On the other hand, data on the bleeding complications were scarce. However, in three of these studies reporting such information, bleeding complications were uncommon and minor.^21,28,49^

The findings of the current meta-analysis showed a relatively high prevalence of DVT and PE in the range of about 30%. Previous relevant meta-analyses have shown a pooled prevalence ranging from about 13%^73,74^ to 20%.^75,76^ However, these have included studies with a large methodological heterogeneity, with the results being dependent on the percentage of the study sample assessed for VTE. Shi et al. showed that the pooled prevalence of PE was increased from 8% to 28% when the assessment was performed in the total population.^77^ In addition, in line with the meta-regression analysis of this study showing that hospitalization in ICU determines a higher prevalence of PE, previous reports have shown a higher pooled prevalence in studies including patients in ICU versus those hospitalized in general wards.^77,78^ Thus, the current analysis reported higher prevalence of VTE compared to the existing literature and this was driven by the methodology of the included studies (screening/assessment in the total sample). The clinical relevance of this methodology is highlighted by the fact that most cases of DVT were reported as asymptomatic in many studies.

It should be noted that the high prevalence of VTE among patients with COVID-19 was observed despite using thromboprophylaxis in the majority of the included studies. In the meta-regression analysis, the prevalence of DVT was lower in studies with mixed dosing of anticoagulation in ⩾ 50% of the population compared to studies with standard prophylactic dosing of anticoagulation in < 50% of the population. A retrospective study in more than 4000 hospitalized patients with COVID-19 showed anticoagulation therapy to be associated with lower mortality and intubation events.^5^ Current recommendations strongly support the use of thromboprophylaxis in all hospitalized patients with COVID-19, although this is based mainly on expert opinion and less so on high-quality evidence.^6–10^ Furthermore, important details such as the optimal dose-intensity of the anticoagulation therapy are lacking.

The findings of this review should be interpreted by considering several limitations. Most important is the heterogeneity among these studies and the lack of information regarding (i) the patients’ individual VTE risk and (ii) details on the anticoagulant therapy (time of initiation, modification, etc.), which might have influenced the outcome. In a sensitivity analysis for identification of publication bias, there was a trend for higher PE prevalence in smaller studies, but there was no apparent trend in DVT prevalence. Furthermore, in a significant proportion of the included studies, exclusive polymerase chain reaction-based diagnosis of COVID-19 was unclear or not reported. Other criteria for diagnosis, such as imaging or other laboratory tests, might have been used but these probably regarded only a minority of patients and not the whole study sample. Thus, the exposure might not have been measured in a strictly reliable way in a minority of patients in some of the studies but this also reflects real clinical practice. However, the outcome was measured in a valid and reliable way in most of the included studies, although, in some of these, adjustment for confounders would be needed for accurate assessment. In addition, meta-regression analysis examined the associations between outcome and several characteristics which were aggregate and summarized at the level of the study, which in turn introduces ecological bias. Last, most of the studies did not provide information on hemorrhagic complications.

Since the screening/assessment process for VTE diagnosis represents a significant source of heterogeneity among such studies, we included only studies that screened/assessed the total population. Limb ultrasonography is an easy test that can be performed massively in the context of a research protocol and can identify asymptomatic patients, which is not an uncommon finding. However, computed tomography pulmonary angiography is performed in selected patients upon clinical suspicion combined with the D-dimer value. By selecting studies that performed these assessments in the whole study population, a more realistic estimate of the DVT/PE prevalence among these patients can be calculated, which in turn determines the pre-test probability in such patients. The latter estimate is a major determinant in a Bayesian approach where the diagnostic strategy depends on the pre-test probability. Thus, the findings of this meta-analysis might provide answers regarding the prevalence of DVT in hospitalized patients with COVID-19, including both symptomatic and asymptomatic cases (the latter being quite common), as well as regarding the prevalence of PE in hospitalized patients with COVID-19 and high suspicion based on clinical characteristics and D-dimer values.

## Conclusion

This systematic review of the evidence suggests that hospitalized patients with COVID-19, who are screened or assessed for VTE, present a pooled prevalence of DVT and PE at about 30% each, and despite thromboprophylaxis in most cases. The VTE risk appears to be considerably higher than in patients without COVID-19 admitted in the same ICUs. Further research is necessary to investigate the individualized VTE risk of patients with COVID-19, the underlying pathogenetic mechanisms, and the optimal preventive anticoagulant therapy.

## Supplemental Material

sj-pdf-1-vmj-10.1177_1358863X21995566 – Supplemental material for Venous thromboembolism in COVID-19: A systematic review and meta-analysis Click here for additional data file. Supplemental material, sj-pdf-1-vmj-10.1177_1358863X21995566 for Venous thromboembolism in COVID-19: A systematic review and meta-analysis by Anastasios Kollias, Konstantinos G Kyriakoulis, Styliani Lagou, Evangelos Kontopantelis, George S Stergiou and Konstantinos Syrigos in Vascular Medicine
