# Supplementary material for: Venous thromboembolism in COVID-19: A systematic review and meta-analysis
Source: Vasc Med. 2021 Apr 4;26(4):415–25. doi: 10.1177/1358863X21995566 (PMC8024143; doi:10.1177/1358863X21995566)

VENOUS THROMBOEMBOLISM IN COVID-19: A SYSTEMATIC REVIEW AND META-ANALYSIS

Supplemental Figure S1. Assessment of the risk of bias of included studies

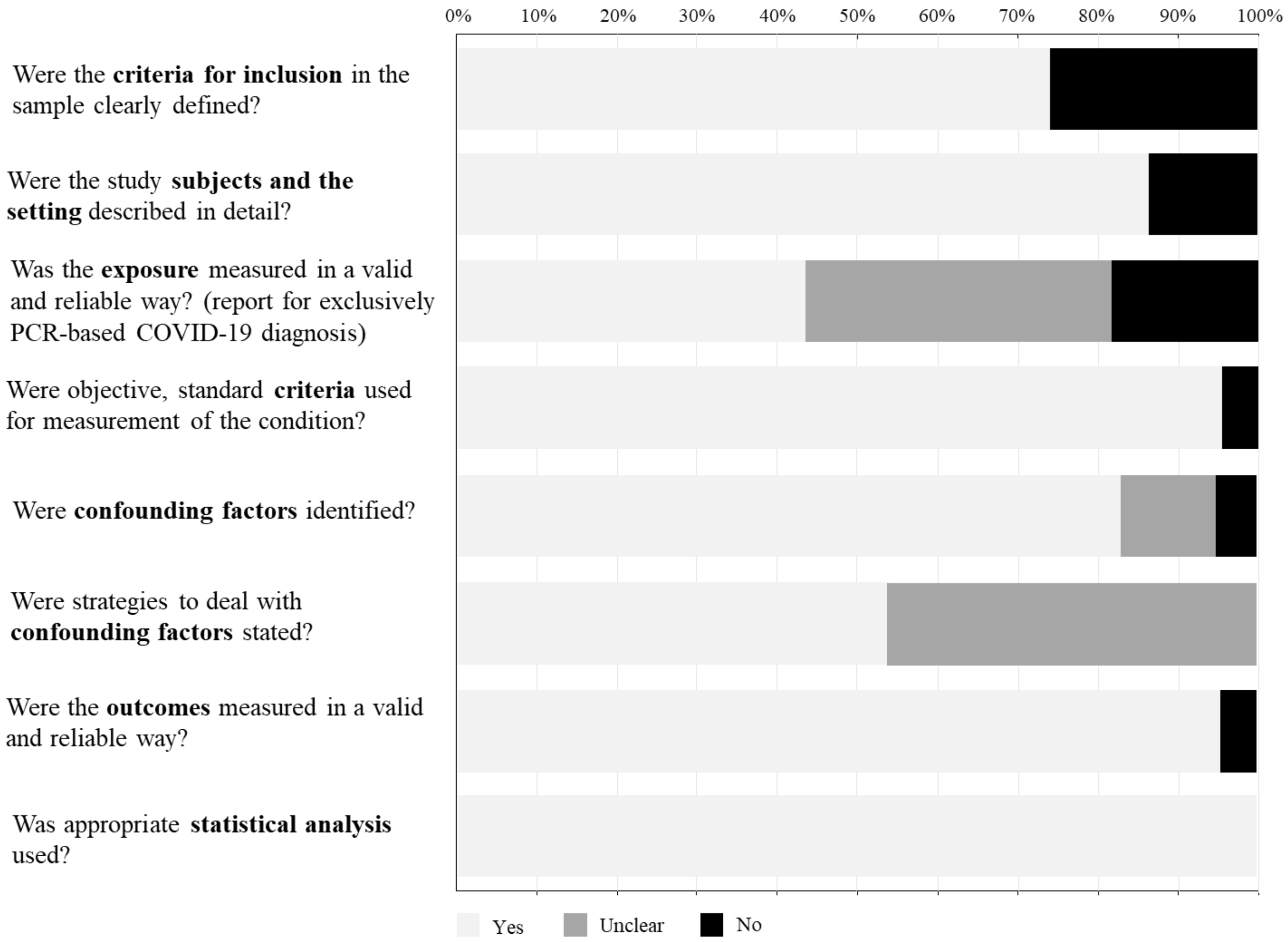

Supplemental Figure S2. Plot of prevalence of pulmonary embolism (A) and deep vein thrombosis (B) versus study sample size

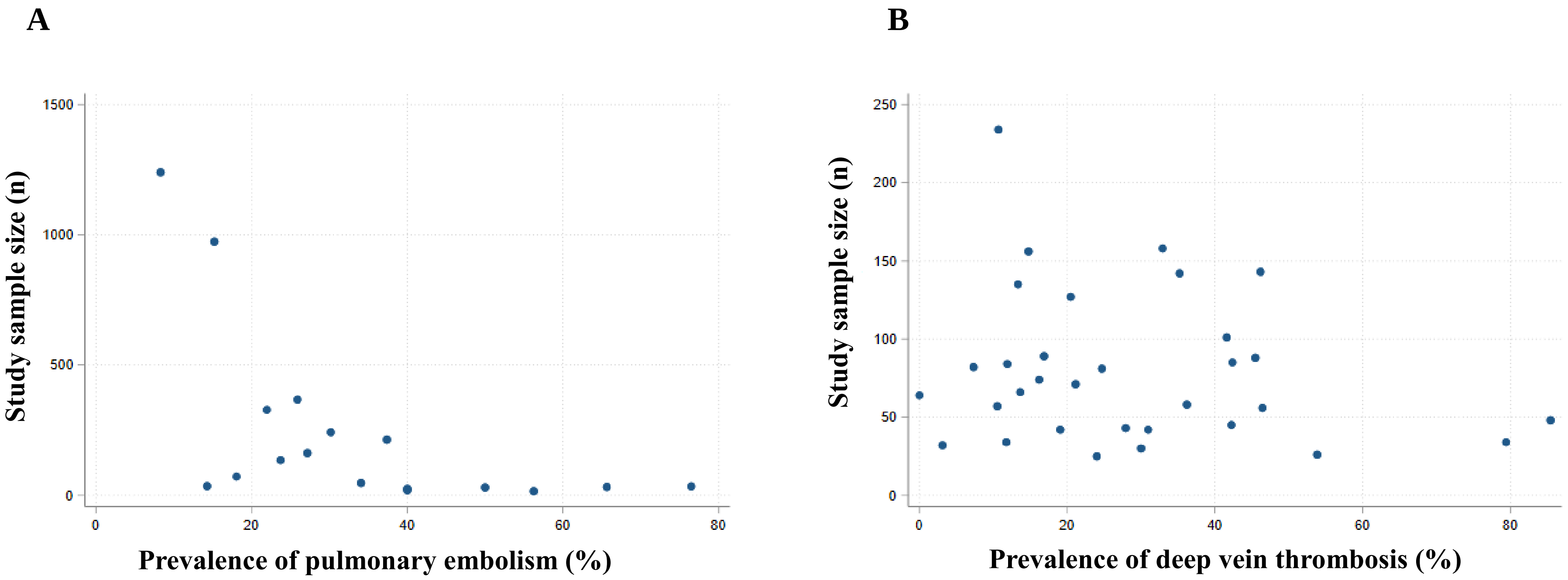

Supplemental Figure S3. Meta-regression bubble plots for the associations of the prevalence of venous thromboembolism

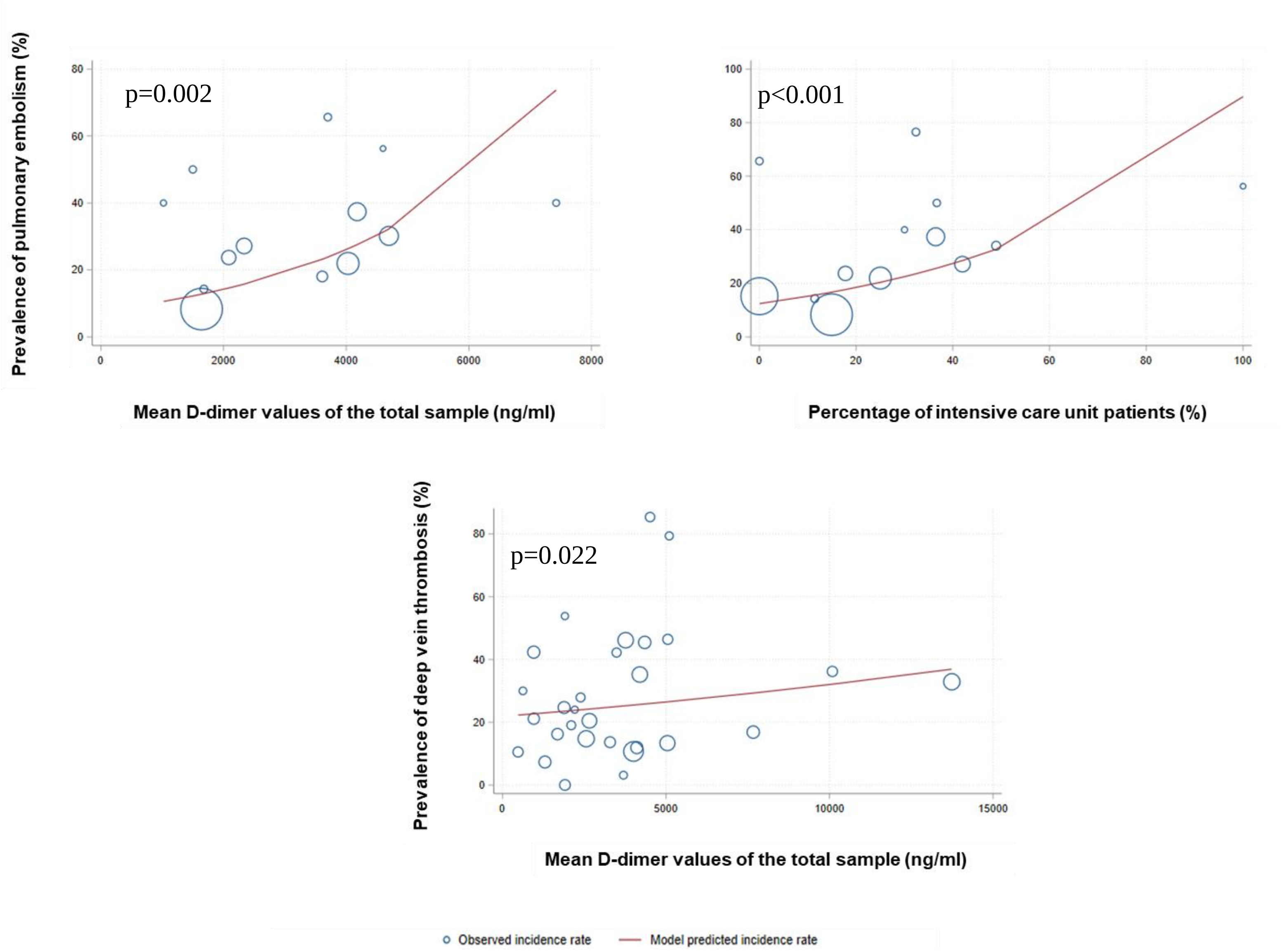

Supplemental Figure S4. Meta-regression bubble plots for the associations of the prevalence of pulmonary embolism after removal of 1 study with extreme values.

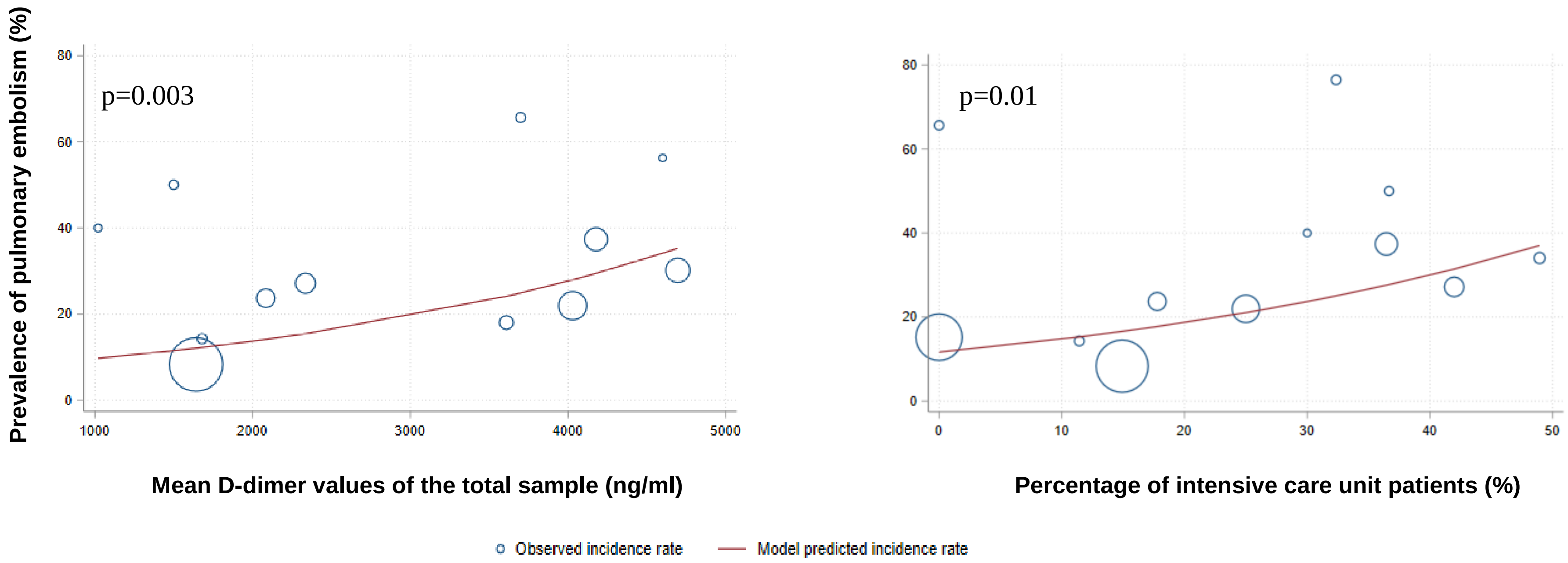

Supplement: sj-pdf-1-vmj-10.1177_1358863X21995566 – Supplemental material for Venous thromboembolism in COVID-19: A systematic review and meta-analysis [file sj-pdf-1-vmj-10.1177_1358863X21995566.pdf]
